# Uremic Toxin Lanthionine Interferes with the Transsulfuration Pathway, Angiogenetic Signaling and Increases Intracellular Calcium

**DOI:** 10.3390/ijms20092269

**Published:** 2019-05-08

**Authors:** Carmela Vigorito, Evgeniya Anishchenko, Luigi Mele, Giovanna Capolongo, Francesco Trepiccione, Miriam Zacchia, Patrizia Lombari, Rosanna Capasso, Diego Ingrosso, Alessandra F. Perna

**Affiliations:** 1 Department of Translational Medical Sciences, University of Campania “Luigi Vanvitelli,” 80131 Naples, Italy; ca.vigorito86@libero.it (C.V.); anishchenkoea@gmail.com (E.A.); giovi.capolongo@gmail.com (G.C.); francesco.trepiccione@unicampania.it (F.T.); miriam.zacchia@unicampania.it (M.Z.); patrizia.lombari@gmail.com (P.L.); 2 Department of Precision Medicine, University of Campania “Luigi Vanvitelli,” 80138 Naples, Italy; r.capasso74@icloud.com (R.C.); diego.ingrosso@unicampania.it (D.I.); 3 Department of Experimental Medicine, University of Campania “Luigi Vanvitelli,” 80138 Naples, Italy; luigi.mele@unicampania.it; 4 Biogem A. C. S. R. L. Contrada Camporeale, 83031 Ariano Irpino AV, Italy

**Keywords:** lanthionine, glutathione, uremic toxin, chronic kidney disease, H_2_S, sulfane sulfur, CBS, CSE, VEGFA, calcium homeostasis

## Abstract

(1) The beneficial effects of hydrogen sulfide (H_2_S) on the cardiovascular and nervous system have recently been re-evaluated. It has been shown that lanthionine, a side product of H_2_S biosynthesis, previously used as a marker for H_2_S production, is dramatically increased in circulation in uremia, while H_2_S release is impaired. Thus, lanthionine could be classified as a novel uremic toxin. Our research was aimed at defining the mechanism(s) for lanthionine toxicity. (2) The effect of lanthionine on H_2_S release was tested by a novel lead acetate strip test (LAST) in EA.hy926 cell cultures. Effects of glutathione, as a redox agent, were assayed. Levels of sulfane sulfur were evaluated using the SSP4 probe and flow cytometry. Protein content and glutathionylation were analyzed by Western Blotting and immunoprecipitation, respectively. Gene expression and miRNA levels were assessed by qPCR. (3) We demonstrated that, in endothelial cells, lanthionine hampers H_2_S release; reduces protein content and glutathionylation of transsulfuration enzyme cystathionine-β-synthase; modifies the expression of miR-200c and miR-423; lowers expression of vascular endothelial growth factor VEGF; increases Ca^2+^ levels. (4) Lanthionine-induced alterations in cell cultures, which involve both sulfur amino acid metabolism and calcium homeostasis, are consistent with uremic dysfunctional characteristics and further support the uremic toxin role of this amino acid.

## 1. Introduction

Lanthionine is a non-protein amino acid generated as a side-product of trassulfuration, a two-step metabolic pathway catalyzed by cystathionine-β-synthase (CBS) and cystathionine-γ-lyase (CSE). Transsulfuration is a bifunctional pathway; in the complete classical mode, the two enzymes act in sequence, where CBS catalyzes the condensation of serine and homocysteine to form cystathionine; this in turn is hydrolyzed by CSE to α-ketobutyrate and cysteine [1,2]. In the second more recently discovered way of action, transsulfuration enzymes may function independently from each other, catalyzing the formation of hydrogen sulfide (H_2_S), the third gaseous vasodilator, mainly from two cysteine molecules and yielding lanthionine as a side product, with a one to one stoichiometric ratio to H_2_S [3].

Lanthionine has been initially considered as a stable indicator of H_2_S production in living systems [4,5]. It has been demonstrated that this amino acid accumulates in circulation in uremia and may inhibit transsulfuration enzymes in cell cultures, possibly contributing to the hyperhomocysteinemia almost constantly associated with uremia [6]. The uremic toxin characteristic of lanthionine has been further assessed in a zebrafish animal model, where it was demonstrated that lanthionine determines significant alterations of larval cardiovascular development, together with behavioral modifications; these alterations are partly reversible upon glutathione treatment [7].

It has been established that H_2_S is present in various forms. The free gaseous form is volatile and difficult to detect, nevertheless the product of H_2_S interactions with other species yields sulfane sulfur, which include persulfides (R-S-SH), polysulfides (R-S_n_-SH or R-S-S_n_-S-R), inorganic hydrogen polysulfides (H_2_S_n_, *n* ≥ 2) and protein-bound elemental sulfur (S^8^). Sulfane sulfur related species are generated following the oxidation of cellular H_2_S and they are thought to serve as a source of endogenously released H_2_S [8,9,10]. Bound sulfane sulfur, rather than free H_2_S, appeared to be altered in uremia [6]. Detection of both gaseous H_2_S and sulfane sulfur is indeed a way to gain information on the effects of lanthionine and its mechanism(s) of action as an uremic toxin, as well as on the general availability of H_2_S in cell cultures [11].

We therefore studied the effects of lanthionine in a human endothelial cell line i) on H_2_S release and sulfane sulfur cellular content; ii) CBS protein content and glutathionylation; iii) the expression of relevant miRNA; iv) the expression of VEGFA and v) intracellular Ca^2+^ levels.

## 2. Results

### 2.1. Lanthionine Decreases H_2_S Release and Sulfane Sulfur Cell Content as well as CBS Protein Levels

In order to measure the effects of lanthionine on the release of H_2_S, as well as on sulfane sulfur content, endothelial EA.hy926 cells were treated with lanthionine, at concentrations comparable with those detected in vivo in uremia [6]. Treatments were also applied under condition maximizing the stimulation of the activity of transsulfuration enzymes, i.e., in the presence of vitamin B_6_, the precursor of pyridoxal phosphate, needed as a coenzyme of both CBS and CSE; cysteine, the substrate for H_2_S biosynthesis; *S*-adenosylmethionine, an allosteric powerful CBS activator.

**Figure 1:**
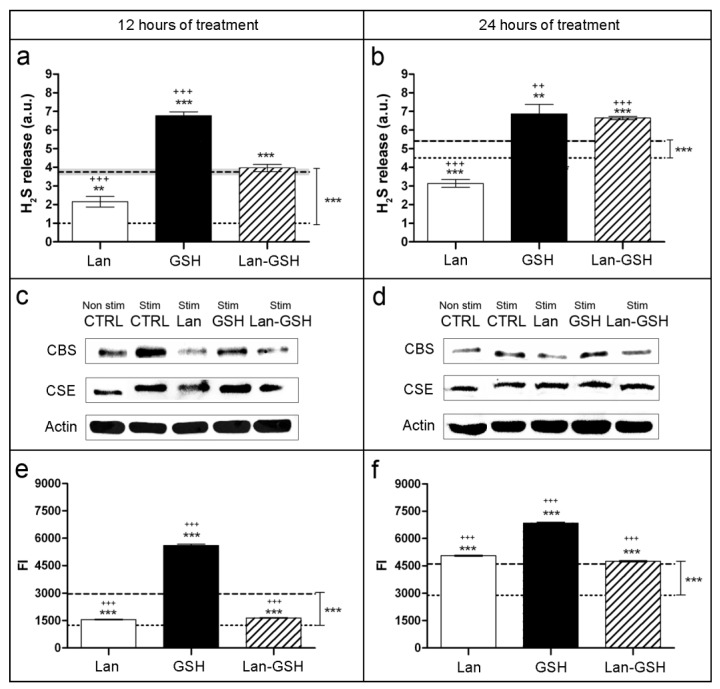
Effects of lanthionine on H_2_S release, sulfane sulfur levels, CBS and CSE protein levels. Cells were incubated with 0.3 μM lanthionine and/or 1 mM GSH, under “stimulated” conditions. H_2_S release in 12 h (**a**) and in 24 h (**b**) is expressed in arbitrary units (a.u.) normalized to the untreated (non-stimulated) control at 12 h, which is made equal to 1. Western blot analysis of CBS and CSE proteins abundance in samples treated during 12 h (**c**) and 24 h (**d**) is shown; β-actin is the loading control. Relative quantity of sulfane sulfur species in endothelial cells, treated for 12 h (**e**) and in 24 h (**f**), were detected using fluorescent probe SSP4 and expressed as fluorescence intensity (FI). In a,b,e,f, dotted lines indicate the average values of untreated controls and bold dashed lines indicate the average values of stimulated control. In all experiments Lan, GSH, Lan with GSH and stimulated controls were incubated with 1 mM Cys, 1 mM vitamin B_6_ and 5 μM SAM, 2 h before treatment started. In a and b the columns represent the mean and error bars indicating the SD from three independent experiments. Gray area behind bold dashed lines represent the SD for stimulated controls; *p* value versus untreated control = ** *p* < 0.01, *** *p* < 0.001, while *p* value versus stimulated control = ^++^ *p* < 0.01, ^+++^ *p* < 0.001 (according to Student’s *t*-test). In e and f, the columns represent the mean and error bars indicate the percent coefficient of variation (%CV) of 10^4^ events; *p* value versus untreated control = *** *p* < 0.001, while *p* value versus stimulated control = ^+++^ *p* < 0.001 (according to Student’s *t*-test). CTRL, control; Lan, lanthionine; GSH, glutathione; CBS, cystathionine-β-synthase; CSE, cystathionine-γ-lyase; Cys, cysteine; SAM, *S*-adenosyl-L-methionine.

Stimulated cell conditions were highly effective to trigger H_2_S release, particularly after the 12 h treatment (Figure 1a). Treatment with lanthionine during 12 h and 24 h both caused a 43% reduction of H_2_S release respect to the stimulated controls (Figure 1a,b). Conversely, glutathione (GSH) treatment induces a 80% and 27% increase of H_2_S release, with respect to the stimulated controls during 12 h and 24 h of treatment, respectively (Figure 1a,b). Lanthionine, in the absence of GSH, exerted a significant long-lasting effect on H_2_S release (Figure 1a,b). After 24 h incubation, the recovery upon lanthionine and GSH combined treatment was prevalent on inhibition of H_2_S release induced by lanthionine alone (Figure 1b). It appears however that the kinetics of the recovery of gaseous H_2_S release and sulfane sulfur increase are quite different upon the addition of both GSH and lanthionine, or GSH alone in the medium, compared to lanthionine treatment. This may reflect the different metabolism of these two sulfur-containing species.

Enzyme protein content was analyzed by Western blotting (Figure 1c,d and Figure S1). It appears that in the samples treated with lanthionine at 12 h CBS is reduced, while CSE is almost unchanged (Figure 1c and Figure S1a,c). In addition, the difference in the intensities of CBS bands in Figure 1c,d is consistent with the apparent difference in the stimulated vs. non-stimulated levels of H_2_S release in Figure 1a,b respectively. This suggests that the stimulation cocktail has a much stronger stabilizing effect on the CBS protein during the shorter incubation range.

In the same cell model, the effect of lanthionine on sulfane sulfur content was evaluated. An inhibitory effect of lanthionine on sulfane sulfur content could only be detected at 12 h incubation with respect to the stimulated sample signal (Figure 1e), while after 24 h incubation, such inhibitory effect of lanthionine on sulfane sulfur levels could no longer be detected (Figure 1f). As expected [10,12], GSH appears to significantly increase sulfane sulfur under stimulated conditions (Figure 1e,f). Combined treatments with lanthionine plus GSH resulted in the almost total abolishment of lanthionine effect on H_2_S release, with respect to GSH alone, at 24 h (Figure 1b). On the other hand, a long lasting effect of lanthionine on sulfane sulfur levels is residual at 24 h, compared with the samples where GSH alone is present (compare Figure 1b,f). This may support the interpretation that higher concentrations of glutathione persulfide (GSSH) may indeed be present in the samples incubated with GSH plus lanthionine, and H_2_S can actually be more easily generated from persulfide degradation in the samples where lanthionine is also present. This is consistent with the notion that sulfane sulfur is lower in high lanthionine uremic serum samples, compared to normal [6].

### 2.2. Lanthionine Hampers CBS Glutathionylation

It has been shown that post-biosynthetic enzymatic glutathionylation of Cys_346_ of CBS significantly increases its activity [13]. In order to check the extent of CBS glutathionylation under our experimental conditions, we processed cell extracts treated with lanthionine and/or GSH. Proteins were immunoprecipitated with a monoclonal anti-GSH antibody and separated by Western blotting, where detection antibody was a monoclonal anti-CBS.

**Figure 2:**
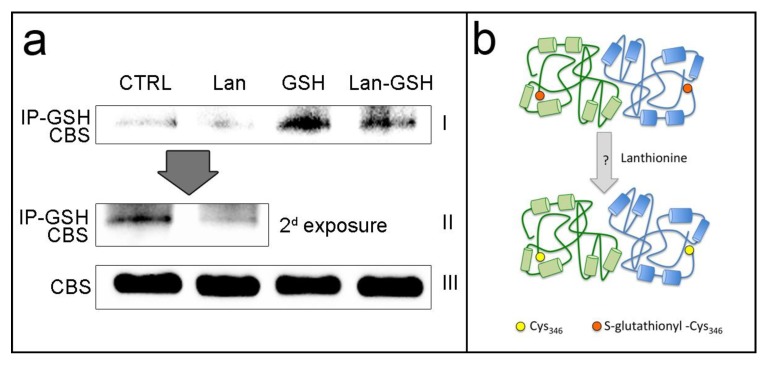
Effect of lanthionine on CBS glutathionylation. (**a**) Endothelial cells were treated with 0.3 μM lanthionine and/or with 1 mM GSH during 24 h. These experiments were accomplished without pre-incubation with the stimulation cocktail, because under stimulating conditions lanthionine significantly reduces the amount of CBS protein, which may interfere with the evaluation of the amount of immunoprecipitated protein. Western blotting shows the analysis of cell protein extracts. First two rows from top (I and II), proteins were immunoprecipitated with anti-GSH antibodies (IP-GSH) and subsequently detected with anti-CBS antibody. Row II: 2nd (prolonged) exposure of the half left part of row I, more clearly showing the difference between lanthionine treated cells and control samples. Row III (CBS); non immunoprecipitated parallel samples treated as in I and II, showing that the initial amounts of CBS was the same in all starting extracts. (**b**) Tentative scheme depicting summarily the possible interpretation of our findings presented in a. According to this model lanthionine lowers CBS activity by hampering CBS activation induced by GSH-dependent glutathionylation. The actual mechanism is not known. Cys, cysteine; CTRL, control; Lan, lanthionine; GSH, glutathione.

Results in Figure 2 and Figure S2 show that lanthionine decreased CBS glutathionylation by about 20%, according to ImageJ analysis. During longer exposure, as shown in Figure 2a, row II, the increased detection sensitivity could reveal, according to ImageJ analysis, 60% decreased glutathionylation caused by lanthionine supplement (inset in Figure S2). GSH, as expected, increased glutathionylation approximately eight folds with respect to the control. Again, the addition of lanthionine to GSH lowered glutathionylation by about 20%.

We interpreted this result as the ability of lanthionine of directly interfering with CBS glutathionylation, possibly by hampering GSH availability at Cys_346_ site. This mechanism can be expected to sum up with respect to lanthionine’s ability to downregulate CBS expression as seen in Figure 1c,d.

### 2.3. Lanthionine is Involved in Fundamental Angiogenic Pathways

**Figure 3:**
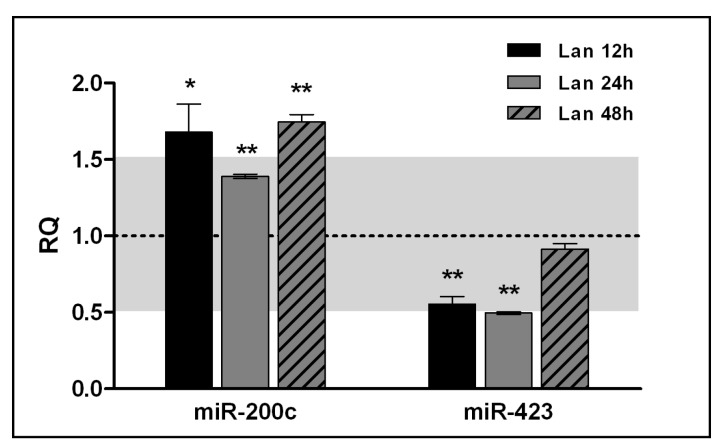
Effect of lanthionine on microRNAs in endothelial cells. Fold changes in miR-200c and miR-423 levels after 12 h, 24 h and 48 h of treatment with 0.3 µM lanthionine in endothelial cells. Untreated control; dashed baseline. The gray area behind the dashed baseline, corresponding, on the *y* axis, to RQ 0.5–1.5, indicates an area of variations of microRNA levels which may be considered physiological with respect to the control. The columns represent the mean and error bars indicating the SD from three independent experiments; *p* values versus untreated control = * *p* < 0.05, ** *p* < 0.01 (according to Student’s *t*-test).

Previous results have shown that lanthionine induces significant changes in the miRNA pattern in zebrafish larvae; these changes were partly comparable to some miRNA modifications detected in uremia [7]. In this work, we undertook a literature search and choose miR-200c and miR-423 since they are altered in kidney disease [14,15,16,17,18,19,20]. Here, we demonstrated that in EAhy926 the expression of miR-200c and miR-423 was affected by lanthionine treatment compared to the untreated control (Figure 3).

In consideration of the implications of uremic toxins in the increased cardiovascular risk, we focused on potential targets of miR-200c and/or miR-423, which may be indeed involved in the pathophysiology of vascular endothelial damage. Our in silico bioinformatics prediction based on the KEGG pathway collection database showed that both miR-200c and miR-423 may exert potential regulatory role in the expression of VEGFA (Table S1). While the role of miR-423 on VEGFA regulation is still uncertain, miR-200c has been described to actually downregulate VEGFA expression in cell cultures [21].

**Figure 4:**
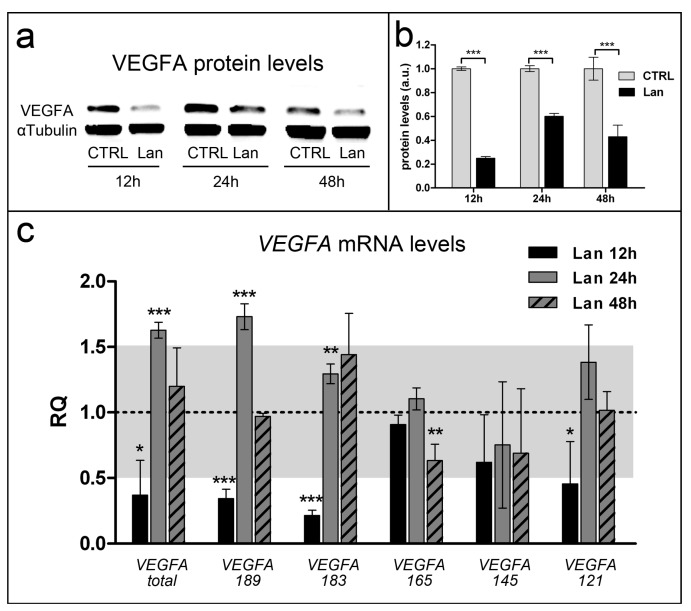
Molecular characterization of intracellular VEGFA levels in endothelial cells after 0.3 µM lanthionine treatment in 12 h, 24 h and 48 h. (**a**) Western blot analysis of VEGFA protein abundance in endothelial cells; αTubulin is the loading control; (**b**) relative differences in protein levels shown in (**a**) were quantitated by using ImageJ software, normalized to αTubulin levels and expressed as arbitrary units (a.u.). (**c**) Relative quantitation (RQ) of the expression levels of VEGFA isoform transcripts (total VEGFA includes all isoforms, VEGFA-121, -145, -165, -183, -189), using qPCR method, in comparison to the untreated control shown as a dashed baseline and made equal to 1. Alterations of mRNA levels, which are being considered physiological with respect to the control, fall within the gray area between 0.5 and 1.5 RQ. The columns in **b** and **c** represent the mean and error bars indicate the SD from three independent experiments; *p* value versus untreated control = * *p* < 0.05, ** *p* < 0.01; *** *p* < 0.001, (according to Student’s *t*-test). CTRL, control; Lan, lanthionine; VEGFA, vascular endothelial growth factor A.

Lanthionine treatment was effective to reduce VEGFA protein levels in a time-dependent fashion (Figure 4a,b). Real-time PCR was used in order to accomplish an extended quantitative analysis of VEGFA transcript levels in endothelial EA.hy926 treated with lanthionine. Results in Figure 4c show that all isoforms could be detected, although isoform 145 was affected by the highest experimental detection variability, likely due to its low expression levels, as described by others [22]. In addition, results indicated that the 12 h treatment with lanthionine determines a general downregulation of VEGFA expression, except in the case of isoform 165 (Figure 4c). Lanthionine treatment at 12 h is consistent with decreased mRNA and protein levels as seen in Figure 4a–c. While longer treatments (24 h and 48 h) showed the lesser inhibitory effect on protein levels, those may be explained by the rebound in transcript levels detected for several isoforms (Figure 4). The system seems to reach a stabilization after 48 h of treatment with lanthionine.

### 2.4. Lanthionine Increases Intracellular Calcium Levels

**Figure 5:**
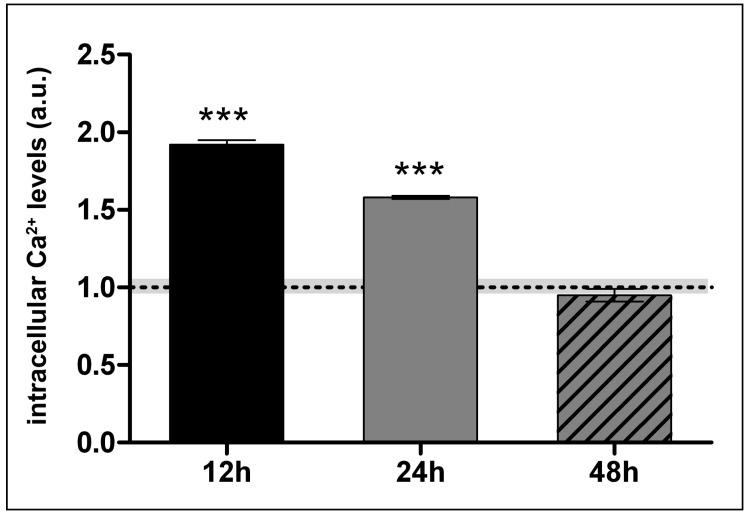
Effect of 0.3 µM lanthionine treatment in 12 h, 24 h and 48 h on intracellular Ca^2+^ levels in endothelial cells. Ca^2+^ levels were detected using the fluorescent probe Fura 2-AM and were expressed as arbitrary units (a.u.), normalized to fluorescence intensity in lanthionine free control cells (dashed line). The columns represent the mean and error bars indicating the SD from three independent experiments; gray area represents the SD for control samples; *p* value versus untreated control = *** *p* < 0.001, (according to Student’s *t*-test). CTRL, control; Lan, lanthionine; Ca^2+^, calcium.

Intracellular calcium levels are important determinants of vascular homeostasis and are connected with both H_2_S and VEGFA functions [23]. We then monitored calcium levels in our system by using a fluorescent specific probe. Lanthionine treatment induced a rise of intracellular calcium ion concentrations, which decayed with time to reach the level of control within 48 h of treatment (Figure 5).

## 3. Discussion

The data presented here deal with three aspects of lanthionine activity as an uremic toxin: (a) the possibility that a reducing microenvironment could relieve lanthionine’s deleterious effects; (b) the way of action of lanthionine on transsulfuration enzymes; (c) the mechanism(s) through which lanthionine may exert its cardiovascular toxicity by interfering with pathways connected to vascular homeostasis.

Lanthionine is a naturally occurring amino acid, produced during the enzymatic biosynthesis of H_2_S from condensation of two cysteine molecules. In vivo, H_2_S is normally produced in many tissues and also the microbiota contributes to its production [24]. One major issue is the way trassulfuration enzymes are regulated in order to partition their activity between H_2_S biosynthesis and the classical trassulfuration, leading to the formation of cysteine from homocysteine. In uremia there is a profound dysregulation of sulfur amino acid metabolism, in that homocysteine is often increased in circulation and also a number of other sulfur-containing intermediates are altered, including *S*-adenosylhomocysteine [25,26]. Lanthionine has been found to accumulate by almost two orders of magnitude in uremic serum. This increase has been related to some important consequences: first, its possible interference with metabolic pathways; second, its possible ability to disrupt tissue and organ function in renal disease. A striking example of the first was the evidence that lanthionine could hamper H_2_S release in cell cultures [6]. Taking into account that transsulfuration enzymes are devoted to both homocysteine metabolism and H_2_S biosynthesis, it should be expected that any inhibitory mechanism could affect both functions. The effect of lanthionine may contribute to an overall impairment of transsulfuration, thus help explaining the presence of a very high prevalence of hyperhomocysteinemia in uremia, which so far has otherwise gone unexplained. As far as the second aspect is concerned, previous evidence showed that such damaging effects do actually take place in zebrafish, although the molecular mechanism was still elusive [7]. Data presented here showed that the induction of a more reducing cellular microenvironment, due to the presence of GSH, tends to hamper lanthionine effects on the transsulfuration enzyme CBS. Moreover, evidence supports a model of action of lanthionine, according to which this amino acid may interfere with the ability of GSH to activate CBS by forming a complex with this enzyme. We found that lanthionine, under stimulating cell conditions, determined a significant reduction of CBS cell content. On the other hand, from the results reported in Figure 2, we could rule out that the decrease of glutathionylation may affect CBS stability in lanthionine-treated cells, because no appreciable changes in CBS levels before immunoprecipitation, could be detected in cells treated with lanthionine compared to control, as well as in the other lanes (Figure 2a row III). On the other hand, we had designed the immunoprecipitation experiment in order to prevent any CBS variations to actually occur and also to avoid confounding due to a CBS protein decrease. Of course, we cannot rule out that in vivo, under stimulating conditions, CBS decrease and reduced glutathionylation may exert a synergic negative effect on the activity of this enzyme.

Our results also showed altered expression levels of miR-200c and miR-423. It has been reported that the same miRNAs are also altered in kidney disease [14]. In addition, target prediction analysis revealed the existence of over 1000 mRNA targets of miR-200c and miR-423, regulating pathways perturbed in various kidney pathologies [14]. These two miRNAs were described as a sensitive (and noninvasive) indicators of kidney damage [15]. The upregulation of miR-200c in inner kidney medulla was observed in 24 h dehydrated mice [16]. Patients with minimal change nephropathy or focal glomerulosclerosis had higher urinary miR-200c levels than those with non-renal diagnoses [17]. miR-200c showed altered expression in various kidney diseases [18]. Renal cortical content of miR-200c was increased with aging [19]. Alb/TGFβ mice overexpress TGF-β and spontaneously develop renal fibrosis and chronic kidney disease (CKD) with age. qRT-PCR studies showed that miR-423-5p was 2.8-fold downregulated in the plasma of Alb/TGFβ mice, confirming the observation from the human studies where levels of miR-423-5p was 2.2-fold downregulated in plasma of patients with estimated glomerular filtration rate (eGFR) < 30 mL/min [20].

Our prediction analysis showed that VEGFA is a target of both miR-200c and miR-423. Trying to understand the potential contribution of lanthionine-induced VEGFA impairment, according to our present data, it should be pointed out that VEGFA is also a potent mitogen for micro- and macro-vascular endothelial cells derived from arteries, veins, and lymphatic vessels [27]. It has been reported that VEGFA inhibition or reduction could result in thrombotic microangiopathy with renal involvement [28,29]. Reduction of VEGFA production by the podocytes may lead to loss of glomerular cells with consequent mesangiolysis. VEGF is required for the proliferation, differentiation, and survival of mesangial and endothelial cells [30].

VEGFA gene encodes for various protein products, with differential cell distribution, solubility or membrane bound characteristics. Based on their amino acids length, the following six isoforms, generated through alternative splicing of the primary transcript, have been described: VEGFA-121, VEGFA-145, VEGFA-165, VEGFA-183, VEGF-189, and VEGFA-206 [31]. VEGFA gene contains eight exons, which are assembled in the final products, so that exons 1 to 5 plus 8 are common to all isoforms. Exon 8 in particular is necessary for the stimulation of mitosis. Then, exon 6 is absent in VEGFA-121 and VEGFA-165; exon 7, which is absent in the isoform 121, contains the heparin-binding site, thus becoming soluble [27,32,33,34]. VEGFA-183 and VEGF-189 are both membrane-bound and differ for only 6 amino acid residues; their functional peculiarities are still partly undetermined [35]. VEGFA-121 and VEGFA-165 are generally the most abundant variants expressed by the examined tissues, followed by VEGFA-189 and VEGFA-183, while VEGFA-145 is detected in small amounts. The expression profile of VEGF splice variants displayed significant heterogeneity between the examined tissues and cell lines [22]. Studies on the structure-function relationships in VEGFA isoforms introduced the concept that they are characterized by different degrees of disorder and flexibility [36].

We found that lanthionine, although generally reducing VEGFA expression, does not affect all isoform expression to the same extent. The affinity for heparin may profoundly affect the bioavailability of VEGF. All VEGFA isoforms, apart from VEGF-121, bind to heparan sulfate or to heparin. VEGF-121 fails to bind to heparin and, therefore, is a freely soluble protein; we found that the expression of this isoform strongly reduced. In contrast, VEGF-189, whose expression is strongly affected by lanthionine, binds to heparin with high affinity and consequently, this isoform is almost completely sequestered in the extracellular matrix. It has been shown that heparin augments the interaction of VEGF-165 with VEGFR2 [37]. VEGF-165 has intermediate properties, as it is secreted and then circulates as a diffusible protein, but a significant fraction remains bound to the cell surface and the extracellular matrix. VEGFA-165 production is not significantly affected by lanthionine treatment. However, the isoforms bound to the extracellular matrix may be released in a diffusible form by heparin, heparinase, or plasmin. Therefore, VEGF-165 may act both as a diffusible factor and as a heparin binding form that is released upon heparin induced cleavage [27]. Heparin may represent indeed an important variable in the uremic microenvironment, influencing VEGF bioavailability in circulation.

One important lanthionine effect is its influence on calcium levels detected in our model system. The mechanism through which lanthionine induces intracellular Ca^2+^ increase is not known. It should be underscored, in this respect, that calcium deposition in the vasculature is a major mechanism contributing to the generation of vascular damage and cardiovascular risk increase in uremia [38]. As far as lanthionine uremic toxicity mechanism is concerned, we found that lanthionine treatment induces an increase of intracellular calcium, while reducing H_2_S release and endogenous sulfane sulfur content. Under these conditions VEGFA expression was reduced, particularly under initial treatment conditions. These results point to a paradoxical behavior, induced by lanthionine in this model, because previous studies showed that VEGF in the short time range stimulates calcium signaling, which was mediated by H_2_S, since this response was inhibited by propargylglycine, a well-known inhibitor of the transsulfuration enzyme CSE [23]. Our results suggest the hypothesis that lanthionine may interfere with the coupling of calcium signaling and VEGF and that this may no longer be tied correctly to H_2_S. The idea that lanthionine may dissociate calcium response to VEGF is also supported by the evidence that VEGF per se is an activator of calcium signaling in brain microvascular cell systems as well as in aortic rings [39]. It should be pointed out, in this respect, that nitric oxide (NO) plays an important role in this interaction. In fact, VEGF activates both eNOS and CSE, thus triggering production of both NO and H_2_S. The two gases, in turn, act synergistically, by activating soluble guanylyl cyclase (NO) and inhibiting phosphodiesterase (H_2_S), respectively [39]. On the other hand, it has been shown that NO plays an inhibitory effects on H_2_S production by CBS [40,41]. At present, we do not have direct information of eNOS activity and NO release in our experimental conditions. However, this aspect may certainly deserve attention in future investigations.

## 4. Materials and Methods

### 4.1. Cell Culture and Treatments

Human EA.hy926 cell line (ATCC) was grown in DMEM medium with 10% Fetal Bovine Serum (S1810-500, Microtech, Pozzuoli, NA, Italy), 2 mM L-glutamine (X0550-100, Microtech, Pozzuoli, NA, Italy) and 0.1% penicillin–streptomycin (L0022-100, Microtech, Pozzuoli, NA, Italy). Cells were grown at 37 °C in a humidified atmosphere with 5% CO_2_.

For H_2_S release detection experiments, cells were plated in cell culture flasks 14 h before any experiment. Experiment was performed in flasks, each containing 600,000 cells. Cells treatments under stimulating conditions (referred to as “stimulated”) were performed by supplying cells in the presence of final concentrations of the following compounds: 1mM DL-cysteine (Cys, 861677, Aldrich, St.Louis, MO, USA), 1 mM pyridoxine hydrochloride (B6, P6280, Sigma, St.Louis, MO, USA) and 5 μM S-adenosyl-L-methionine (SAM, B9003S, New England Biolabs Inc, Ipswich, MA, USA). Cys-B6-SAM “stimulated” pre-treatment was initiated 2 h before the main treatment procedure. The latter consisted of cell treatment with 1 mM reduced L-glutathione (GSH, G6013, Sigma, St.Louis, MO, USA) and/or 0.3 μM DL-lanthionine (Lan, L0010, TCI, Tokyo, Japan). The concentration of lanthionine was based on the actual concentration detected in uremic serum [6]. DL-lanthionine solubility is 50 mg/mL in 1 M HCl (240 mM). A 10^5^ fold dilution of the lanthionine stock in DMEM was prepared. The effect of solvent, in terms of HCl addition to DMEM could be indeed considered negligible, also by considering that neither pH variation nor effect on cell vitality could be detected.

To study VEGFA and miRNA expression, cells were treated with 0.3 μM lanthionine during 12, 24 and 48 h. Cells were grown in six well plates, 200,000 cells per well.

For intracellular calcium levels measurements, cells were treated with 0.3 μM lanthionine during 12 h, 24 h and 48 h. Cells were grown in 24 well plates, 30,000 cells per well.

### 4.2. H_2_S Detection and Quantitation

H_2_S detection was performed using the method LAST [11]. This method, based on lead acetate test strips (06728, Sigma-Aldrich, St.Louis, MO, USA), detects H_2_S on contact with lead acetate forming a black precipitate, indicated by a visible black-colored reaction on the paper strip. In brief, ø 4 mm sterile lead-containing paper strips were attached to the inner side of the lids of cell culture flasks, in order to become directly exposed to H_2_S release during treatments. At the end of incubation (12 h and 24 h of exposure) lead-containing paper strips were scanned and analyzed using imageJ software (National Institutes of Health, Bethesda, MD, USA) to quantitate the intensity of black-stained precipitates. The amount of released H_2_S was normalized in arbitrary units in comparison to untreated control (CTRL) after 12 h of treatment. Samples were always analyzed in triplicate. Results represent the mean and SD of three independent biological repeats.

### 4.3. Sulfane Sulfurs Detection

Sulfane sulfurs detection in EA.hy926 cells was performed using a fluorescent Sulfane Sulfur Probe 4 (SSP4; SB10, Dojindo Laboratories, Kumamoto, Japan). Reactions were performed as previously described [11]; in brief, cells were incubated with 1.5 μM SSP4 probe in serum free Dulbecco’s Modified Eagle’s Medium without Phenol Red (DMEM; D4947, Sigma, St.Louis, MO, USA) with 150 μM cetyl-trimethylammonium bromide (CTAB; H9151, Sigma, St.Louis, MO, USA) for 15 min at 37 °C in a humidified atmosphere with 5% CO_2_, afterwards cells were washed twice with PBS. Fluorescence was measured using flow cytometry (Accuri™ C6 Plus, BD Biosciences, San Jose, CA, USA), in the green channel (FITC). Experiments were performed in triplicate and data were analyzed using the GraphPad Prism software.

### 4.4. Measurement of the Intracellular Ca^2+^ Concentration Ratio

Measurement of Ca^2+^ in EA.hy926 cells was performed using a fluorescent Fura 2-AM probe (F1201, ThermoFisher Scientific, Milan, Italy). Reactions were performed according to the supplier’s protocol. Briefly, cells were washed twice in PBS and incubated with 3 μM probe in PBS for 30 min at 37 °C in a humidified atmosphere with 5% CO_2_. At the end of incubation cells were washed with PBS. Then, the fluorescence intensity was detected at λ_Ex_340 nm and λ_Em_510 nm by a fluorescence multi-reader (Infinite 200, Tecan Trading AG, Männedorf, Switzerland). Experiments were performed in triplicate and the data were analyzed using GraphPad Prism software.

### 4.5. Protein Extraction

Proteins were extracted using RIPA buffer (TCL131, HiMedia, Mumbai, India) containing a cocktail of protease inhibitors (11836153001, Roche, Milan, Italy). Protein concentrations were determined according to Bradford (Bradford Protein Assay Kit; 5000001, BioRad, Hercules, CA, USA). Samples were stored at −20 °C in preparation for Western Blot analysis.

### 4.6. Immunoprecipitation

The immunoprecipitation was performed in order to detect GSH/CBS interaction in EA.hy926 cells, treated during 24 h with 1 mM GSH and/or 0.3 lanthionine as described in Section 4.1. This experiment was performed using Immunoprecipitation Kit—Dynabeads™ Protein G (00330770, Thermo Fisher Scientific, Vilnius, Lithuania), according to the manifacturer’s protocol. Briefly, 100 μg of proteins were incubated with Dynabeads crosslinked with the relevant antibody (7 μg of anti-GSH, ab19534, Abcam, Cambridge, MA, USA) followed by denaturing elution. Immunoprecipitated samples were loaded onto the gel and analyzed by Western blotting.

### 4.7. Western Blotting Analysis

Proteins were electrophoretically separated on precast gels 8–16% (456-1104, mini-PROTEANS^®^ TGX™ Gels, Bio-Rad, Hercules, CA, USA) and transferred onto 0.2 μm nitrocellulose membrane (1704158, Trans-Blot^®^ Turbo™, Bio-Rad, Hercules, CA, USA). Protein detection was performed using the following primary antibodies: anti-CBS (ab140600, Abcam, Cambridge, MA, USA), anti-CSE (ab136604, Abcam, Cambridge, MA, USA), anti-VEGFA (E-AB-11647, Elabscience, Houston, TX, USA). Anti-Actin (E-AB-20031, Elabscience, Houston, TX, USA) and anti-Tubulin (2125S, Cell Signalling, Leiden, WZ, The Netherlands) were employed as loading controls as appropriate. Secondary antibodies anti-rabbit (NC27606, Immunoreagents Inc., Raleigh, NC, USA) and anti-mouse (Immunoreagents Inc., Raleigh, NC, USA) conjugated with horseradish peroxidase were utilized. Detection of the immunocomplexes was obtained by chemiluminescence, utilizing Immobilon Western Chemiluminescent HRP Substrate (WBKLS0500, Millipore Corporation, Billerica, MA, USA) and transilluminator (ChemiDoc™, Bio-Rad, Hercules, CA, USA). Signal intensity was quantified with ImageJ software.

### 4.8. RNA Extraction

mRNA and miRNA were extracted from cells using mirVana™ PARIS™ kit (AM1556, ThermoFisher Scientific, Milan, Italy) according to the supplier’s protocol. RNA concentration was measured by means of NanoDrop UV/Vis micro-spectrophotometry (ND-1000; NanoDrop Technologies, Wilmington, DE, USA). Samples were stored at −80 °C.

### 4.9. Reverse Transcription, PCR and qPCR

cDNA was synthesized from 1 μg of total RNA. For reverse transcription, the QuantiTect^®^ reverse transcription kit and gDNA wipeout (205313, Qiagen, Hilden, Germany) were used according to the supplier’s protocol. Reactions were performed in Veriti^®^ 96-Well Thermal Cycler (Applied Biosystems, Foster City, CA, USA). cDNA concentration was measured by means of NanoDrop UV/Vis micro-spectrophotometry. cDNA samples were stored at −20 °C.

qPCR experiments were performed using 200 ng cDNA, Power SYBR™ Green PCR Master Mix (4367659, ThermoFisher Scientific, Milan, Italy) and ViiaTM7 Thermal Cycler (Applied Biosystems, Foster City, CA, USA). For total VEGFA amplification, primers were used as according to Medford et al. [34]; VEGFA isoforms -121, -145, -165, -189 were amplified using primer oligonucleotide sequences as described by Wang et al. [33]; VEGFA-183 isoform was amplified using the following primers: forward 5′- GCA AGA AAT CCC GTC CCT GTG G -3′ and reverse 5′- TCA CCG CCT CGG CTT GTC ACA T -3′; Amplification conditions were the following: 95 °C for 15 min, followed by 40 cycles of 94 °C for 15 s, annealing step was carried out at 61 °C for 30 s and 72 °C for 30 s. To determine the relative quantities of the genes of interest, transcripts present in the various experimental conditions were compared with control. 18S or GAPDH (ThermoFisher Scientific, Milan, Italy), as appropriate, were used as housekeeping genes for all real-time PCR experiments, to normalize the results of the detected transcript levels. Data were handled by Real-Time PCR System ViiaTM7 software. ΔΔ*C*t calculations were also manually verified (“Users bulletin”, ABI PRISM 7700 Sequence Detection System 1997).

### 4.10. miRNA Profiling

miRNA expression was determined using samples obtained from untreated control EA.hy926 cells and cells exposed with 0.3 μM lanthionine during 12–48 h. miRNA were retro-transcribed with RT-TaqMan^®^ MicroRNA Assays: miR-200c (RT/TM 002300), miR-423 (RT/TM 002340) (Applied Biosystems, Pleasanton, CA, USA) on Veriti^®^ 96-Well Thermal Cycler, under the following conditions: 16 °C for 30 min, 42 °C for 30 min, 85 °C for 5 min. Subsequently, cDNA were amplified using corresponding TaqMan^®^ MicroRNA Assays (4366596, Applied Biosystems, Vilnius, Lithuania) on a ViiaTM7 Thermalcycler under the following conditions: 50 °C for 2 min, 95 °C for 10 min and 95 °C for 15 s, 60 °C for 1 min followed by 40 cycles. Relative quantification was performed using the ΔΔCt method using U6 snRNA (RT/TM 0019732, Applied Biosystems, Pleasanton, CA, USA) as a housekeeping gene product. Differential levels of each miRNA expressed were evaluated by the ViiaTM7 software.

### 4.11. Bioinformatics Analysis

The information about pathways involving gene targets for miRNA-200c and miRNA-423 was obtained using the miRSystem software (ver. 20160513-miRNAsystem.cgm.ntu.edu.tw). In order to draw inferences on the potential functional interactions between miRNA and their gene targets, pathways identified were listed according to the KEGG (Kyoto Encyclopedia of Genes and Genomes) pathway map. Relevant nomenclature consists of a molecular network in terms of the KEGG Orthology (KO) groups. miRNA genes targets analysis includes genes selected according to the relevance to processes involved in kidney disease.

### 4.12. Statistical Analysis

Unpaired, two tailed, Student’s t-test was utilized to compare means, as appropriate (means were considered significantly different as *p* < 0.05). Data are expressed as the means ± standard deviation (SD), except where otherwise specified. Results were analyzed using the statistics software GraphPad Prism Version 5.0 (GraphPad Software, San Diego, CA, USA).

## 5. Conclusions

Lanthionine reduced the H_2_S release from cell cultures as well as sulfane sulfur, indicating that this amino acid may contribute to the reduction of H_2_S in uremia. This effect may be at least partly mediated by both CBS downregulation and a reduction of its glutathionylation, which is a known enzyme activating post-biosynthetic modification. These effects are susceptible to partial recovery upon co-incubation with GSH, thus underscoring the relevance to uremia, a condition characterized by oxidative stress and, hence, inflammation. Lanthionine exerted a calcium ionophore role in our system, which was accompanied by an acute inhibitory effects towards the expression of VEGFA. This is likely mediated by an upregulation of miR-200c, which, in fact, is increased in our model upon lanthionine treatment. It has been described that miR-200c stimulates H_2_S production and exerts an anti-oncogene role. Lanthionine-induced alterations in cell cultures, which span over various aspects including sulfur amino acid metabolism and calcium homeostasis, further support the uremic toxin role of this amino acid.

## Supplementary Materials

Supplementary materials can be found at https://www.mdpi.com/1422-0067/20/9/2269/s1.

## Abbreviations

AdoMet	*S*-adenosyl-*L*-methionine
B_6_	Pyridoxalphosphate
Ca^2+^	Calcium
CBS	Cystathionine-β-synthase
CSE	Cystathionine-γ-lyase
CTRL	Control
Cys	Cysteine
GSH	Glutathione
H_2_S	Hydrogen sulfide
Lan	Lanthionine
LAST	Lead Acetate Strip Test
qPCR	quantitative Polimerase Chain Reaction
SSP4	Sulfane Sulfur Probe 4
VEGF	Vascular Endothelial Growth Factor
