# Supplementary material for: Uremic Toxin Lanthionine Interferes with the Transsulfuration Pathway, Angiogenetic Signaling and Increases Intracellular Calcium
Source: Int J Mol Sci. 2019 May 8;20(9):2269. doi: 10.3390/ijms20092269 (PMC6539355; doi:10.3390/ijms20092269)
Supplement: Supplementary file 1 [file ijms-20-02269-s001.zip › ijms-484747-proofback-suppl/ijms-484747-suppl-figures_07-05-2019.docx]

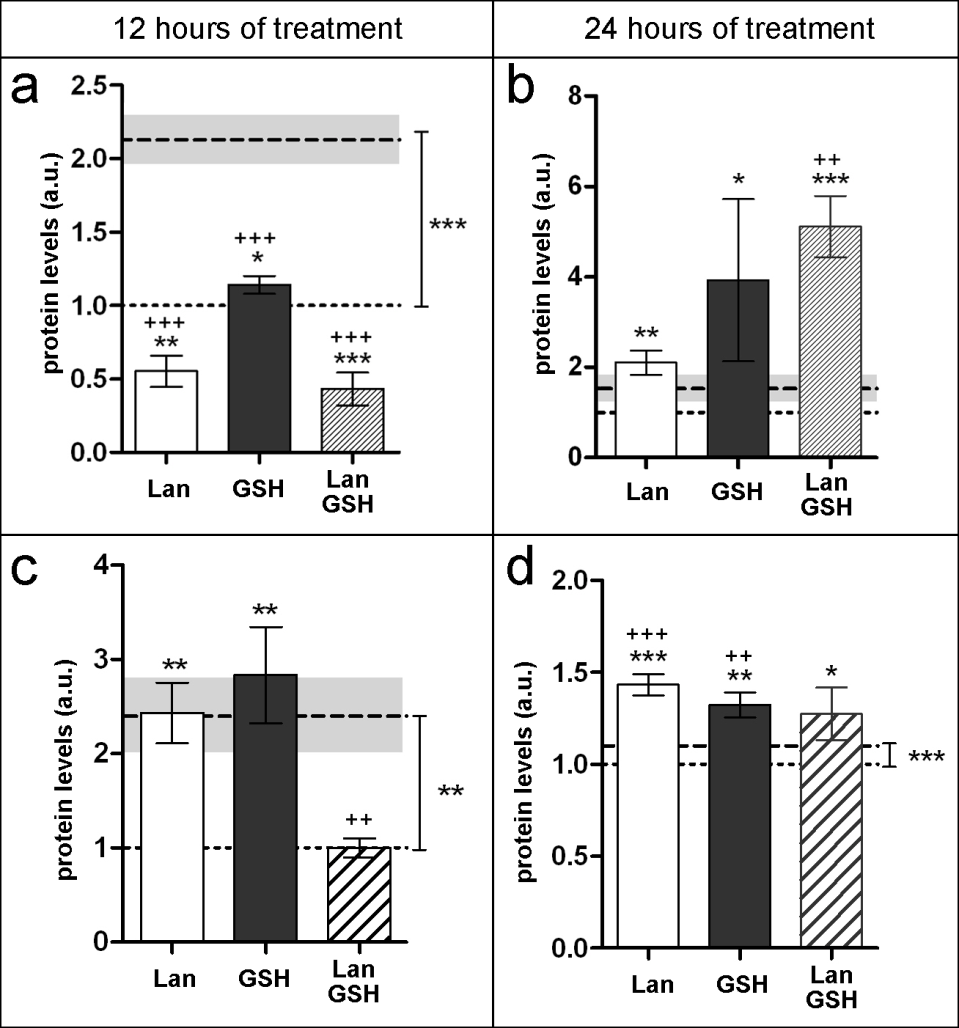


**Supplemental material. Figure S1.** Densitometric analyses of CBS and CSE protein levels. Western blot analysis of CBS and CSE protein abundance in endothelial cells incubated with 0.3 μM lanthionine and/or 1 mM GSH, under “stimulated” conditions during 12 h and 24 h, as explained in Figure 1. Diagrams **a** and **b** show relative differences of CBS levels; diagrams **c** and **d** show relative differences of CSE levels. Both proteins were analyzed after 12 h (**a,c**) and after 24 h (**b,d**) of treatment. All data are normalized to β-actin levels, measured using ImageJ software and expressed in arbitrary units (a.u.). Columns represent the mean and error bars indicating the SD from three independent experiments. Each experiment was carried out and Western blot developed as the ones reported as an example in **Figure 1c,d**. Dotted lines indicate the average values of untreated controls and bold dashed lines indicate the average values of stimulated control. Gray area behind bold dashed lines represent the SD for stimulated controls; *p* value versus untreated control = *p<0.05, **p<0.01, ***p<0.001, while *p* value versus stimulated control = ^++^p<0.01, ^+++^p<0.001 (according to Student’s t-test). CTRL, control; Lan, lanthionine; GSH, glutathione; CBS, cystathionine-β-synthase; CSE, cystathionine-γ-lyase; stimulated conditions; 1 mM cysteine, 1 mM vitamin B_6_, 5 µM *S*-adenosyl-L-methionine.


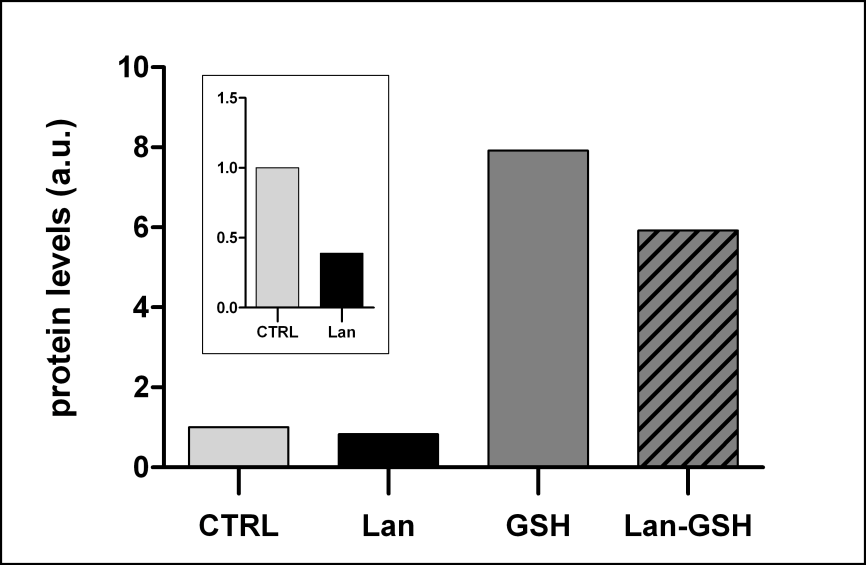


**Supplemental material. Figure S2.** Densitometric analyses of glutathionylated CBS protein levels. Endothelial cells were incubated with 0.3 μM lanthionine and/or 1 mM GSH during 24 h. Western blotting analysis of cell protein extracts immunoprecipitated with anti-GSH antibodies and subsequently detected with anti-CBS antibody, are shown in **Figure 2a**. The diagram here shows relative differences of CBS levels corresponding to row I in **Figure 2a**, measured using ImageJ software and expressed as arbitrary units (a.u.). Inset shows relative differences of CBS levels detected, in the same membrane, after a second prolonged exposure (to better show differences between CTRL and Lan samples) during chemiluminescence analysis and corresponding to row II in **Figure 2a**. CTRL, control; Lan, lanthionine; GSH, glutathione; CBS, cystathionine-β-synthase.
