# Supplementary material for: Uremic Toxin Lanthionine Interferes with the Transsulfuration Pathway, Angiogenetic Signaling and Increases Intracellular Calcium
Source: Int J Mol Sci. 2019 May 8;20(9):2269. doi: 10.3390/ijms20092269 (PMC6539355; doi:10.3390/ijms20092269)
Supplement: Supplementary file 1 [file ijms-20-02269-s001.zip › ijms-484747-proofback-suppl/Table S1_07-05-2019.docx]

**Supplemental material. Table S1.** KEGG pathways involving miR-200c and miR-423. Pathways involve gene targets for miR-200c and miR-423, according to miRSystem software (ver. 20160513-miRNAsystem.cgm.ntu.edu.tw). In order to draw inferences on potential functional interactions between miRNA and their gene targets, pathways identified are listed according to the KEGG (Kyoto Encyclopedia of Genes and Genomes) pathway map. Relevant nomenclature consists of a molecular network in terms of the KEGG Orthology (KO) groups. miRNA genes targets analysis includes genes selected according to the relevance to processes particularly involved renal disease and other pathologies.

|  | **PATHWAY** | **miR-200c** | **miR-423** |
| --- | --- | --- | --- |
| ECM FORMATION AND CELL JUNCTION | **ECM-RECEPTOR INTERACTION** | *COL4A1, DAG1* |  |
|  | **CELL ADHESION MOLECULES (CAMS)** | *OCLN, SDC2* |  |
|  | **ADHERENS JUNCTION** | *YES1, SMAD2, SMAD4,CDC42* |  |
|  | **TIGHT JUNCTION** | *AKT2, KRAS, OCLN, CDC42, YES1* |  |
|  | **FOCAL ADHESION** | *VEGFA, COL4A1, PRKCA, RAC1, CDC42, MYLK, MAPK9* | *VEGFA, RAC1* |
| METABOLISM | **GLUTATHIONE METABOLISM** | *GSTA4* |  |
|  | **CYSTEINE AND METHIONINE METABOLISM** | *DNMT3A, DNMT3B, ENOPH1* |  |
|  | **ADIPOCYTOKINE SIGNALING PATHWAY** | *PRKAA2, AKT2, MAPK9, IKBKB, IRS2, ADIPOR2* | *ADIPOR2* |
| FUNDAMENTAL REGULATORY/SIGNAL TRANSDUCTION PATHWAYS | **MAPK SIGNALING PATHWAY** | *PRKACB, CACNB4, KRAS, PRKCA, CACNA1C, AKT2, CDC42, MAPK9, FGF23, RAC1, MAP3K1* | *FGFR2, RAC1* |
|  | **UBIQUITIN MEDIATED PROTEOLYSIS** | *MAP3K1, UBE2W* |  |
|  | **WNT SIGNALING PATHWAY** | *PRKACB, SMAD2, PRKCA, RAC1, MAPK9* | *RAC1* |
|  | **MTOR SIGNALING PATHWAY** | *VEGFA, PDPK1, RPS6KB1, AKT2, PRKAA2* | *VEGFA* |
|  | **PHOSPHATIDYLINOSITOL SIGNALING SYSTEM** | *PIP4K2B, PTEN, PRKCA, ITPR2* |  |
| RENAL AND CARDIOVASCULAR FUNCTION | **CARDIAC MUSCLE CONTRACTION** | *ATP2A2, CACNB4* |  |
|  | **DILATED CARDIOMYOPATHY** | *DAG1, ATP2A2, DMD, PRKACB, CACNB4* |  |
|  | **ARRHYTHMOGENIC RIGHT VENTRICULAR CARDIOMYOPATHY (ARVC)** | *DMD, DAG1, ATP2A2, CACNB4, CACNA1C* |  |
|  | **VASCULAR SMOOTH MUSCLE CONTRACTION** | *PPP1R12B, PRKACB,ENDRA, ITPR1, ITPR2, MYLK* |  |
|  | **HYPERTROPHIC CARDIOMYOPATHY (HCM)** | *DMD, ATP2A2, CACNB4, DAG1* |  |
|  | **VEGF SIGNALING PATHWAY** | *VEGFA, PRKCA, RAC1, KRAS, CDC42, AKT2* | *VEGFA, RAC1* |
|  | **VASOPRESSIN-REGULATED WATER REABSORPTION** | *PRKACB* |  |
|  | **LEUKOCYTE TRANSENDOTHELIAL MIGRATION** | *OCLN, RAC1, PRKCA* | *RAC1* |
|  | **TYPE II DIABETES MELLITUS** | *SOCS4, IRS1, IRS2* |  |
|  | **INSULIN SIGNALING PATHWAY** | *SOCS4, PDPK1, IRS1, PRKAG2, KRAS, AKT2, MAPK9* |  |
|  | **ALDOSTERONE-REGULATED SODIUM REABSORPTION** | *IRS1, IRS2, PDPK1, KRAS, PRKCA* |  |
| CANCER | **P53 SIGNALING PATHWAY** | *PTEN, CCNE2, CDK2, SIAH1* |  |
|  | **PATHWAYS IN CANCER** | *VEGFA, BCL2, SMAD2, SMAD4, RAC1, PTEN, PRKCA, KRAS, CDKN1B, COL4A1, FGF23, FN1, AKT2, CDC42, MAPK9* | *FGFR2, RAC1* |
|  | **BLADDER CANCER** | *VEGFA, KRAS* | *VEGFA* |
|  | **RENAL CELL CARCINOMA** | *VEGFA, RAC1, KRAS, AKT2, CDC42, PAK7, PAK2, RAP1B* | *VEGFA, RAC1* |
| IMMUNE SYSTEM | **CHEMOKINE SIGNALING PATHWAY** | *PRKACB, RAC1, KRAS, CDC42, AKT2* | *RAC1* |
|  | **CYTOKINE-CYTOKINE RECEPTOR INTERACTION** | *TNFRSF11B, VEGFA, TNFSF8* | *VEGFA* |
|  | **TGF-BETA SIGNALING PATHWAY** | *SMURF2, SMAD2, SMAD4, SP1* |  |
